# Supplementary material for: Association of RASGRP1 polymorphism with vascular complications in Chinese diabetic patients with glycemic control and antihypertensive treatment
Source: Cardiovasc Diabetol. 2024 May 10;23:166. doi: 10.1186/s12933-024-02267-2 (PMC11088008; doi:10.1186/s12933-024-02267-2)

**Supplementary table1. Distribution of RASGRP1 genotype and allele frequency among different treatment groups under different treatment models**

|  | Genotype | Active (%) | Placebo (%) | χ2 | *P* | Intensive (%) | Standard (%) | χ2 | *P* |
| --- | --- | --- | --- | --- | --- | --- | --- | --- | --- |
| rs56254815 | AA | 423(63.2) | 425(61.8) | 0.310 | 0.851 | 447(65.0) | 401(59.9) | 4.138 | 0.124 |
|  | AG | 223(33.3) | 238(34.6) |  |  | 216(31.4) | 245(36.6) |  |  |
|  | GG | 23(3.4) | 25(3.6) |  |  | 25(3.6) | 23(3.4) |  |  |
| rs12593201 | GG | 235(35.1) | 255(37.1) | 0.666 | 0.719 | 237(34.4) | 253(37.8) | 2.636 | 0.269 |
|  | GA | 333(49.8) | 328(47.7) |  |  | 350(50.9) | 311(46.5) |  |  |
|  | AA | 101(15.1) | 105(15.3) |  |  | 101(14.7) | 105(15.7) |  |  |
| rs7403531 | CC | 251(37.5) | 266(38.7) | 0.792 | 0.678 | 250(36.3) | 267(39.9) | 2.794 | 0.249 |
|  | CT | 335(50.1) | 329(47.8) |  |  | 352(51.2) | 312(46.6) |  |  |
|  | TT | 83(12.4) | 93(13.5) |  |  | 86(12.5) | 90(13.5) |  |  |
|  | Total | 669(49.3) | 688(50.7) |  |  | 688(50.7) | 669(49.3) |  |  |

**Supplementary table2. The baseline clinical characteristics of patients under the hypoglycemic treatment mode and the antihypertensive treatment model∗.**

| **Characteristic** | **Glucose control cohort** | | **Blood pressure lowering cohort** | |
| --- | --- | --- | --- | --- |
|  | **Intensive (*N* = 688)** | **Standard (*N* = 669)** | **Active (*N* = 669)** | **Placebo (*N* = 688)** |
| Male sex, n (%) | 345(50.1%) | 359(53.7%) | 330(49.3%） | 374(54.4%） |
| Age(yr), mean (SD) | 65±6 | 65±6 | 65±6 | 65±6 |
| Age when diabetes first diagnosed(yr), mean (SD) | 57±8 | 57±8 | 57±8 | 57±8 |
| Duration of diabetes, mean (IQR) | 8(3-12) | 8(3-11) | 8(3-11) | 8(3-11) |
| **Blood-pressure assessment** | | | | |
| Systolic blood pressure(mmHg), mean (SD) | 139.3±21.3 | 139.9±20.3 | 140.4±21.6 | 138.8±19.9 |
| Diastolic blood pressure(mmHg), mean (SD) | 78.6±10.9 | 78.0±11.0 | 78.6±11.1 | 78.0±10.8 |
| **Blood-glucose assessment** | | | | |
| Glycated hemoglobin (%), mean (SD) | 7.6±1.7 | 7.7±1.8 | 7.6±1.8 | 7.6±1.6 |
| Fasting blood glucose (mmol/L), mean (SD) | 8.6±2.9 | 8.5±2.9 | 8.8±3.1 | 8.4±2.7 |
| **Other major risk factors assessment** |  |  |  |  |
| BMI (kg/m2), mean (SD) | 25.4±3.2 | 25.1±3.0 | 25.2±3.2 | 25.3±3.0 |
| Serum creatinine (umol/l), mean (SD) | 80.0±25.4 | 81.9±36.3 | 79.9±24.1 | 81.9±36.9 |
| Urinary albumin: creatinine(mg/mmol), median (IQR) | 14.7(1.2-7.1) | 20(1.1-6.9) | 17.1(1.1-8.0) | 17.5(1.1-6.2) |
| Total cholesterol(mmol/l), mean (SD) | 5.3±1.2 | 5.4±1.2 | 5.4±1.2 | 5.3±1.2 |
| High-density lipoprotein(mmol/l), mean (SD) | 1.3±0.4 | 1.3±0.4 | 1.3±0.4 | 1.3±0.4 |
| Low-density lipoprotein(mmol/l), mean (SD) | 3.5±5.3 | 3.2±1.0 | 3.5±5.4 | 3.2±1.0 |
| Triglyceride(mmol/l), mean (SD) | 2.0±2.0 | 2.1±1.8 | 2.1±2.1 | 2.0±1.8 |
|  |  |  |  |  |
| **Continued to Supplementary table2. The baseline clinical characteristics of patients under the hypoglycemic treatment mode and the antihypertensive treatment model∗.** | | | | |
| **Characteristic** | **Glucose control cohort** | | **Blood pressure lowering cohort** | |
|  | **Intensive (*N* = 688)** | **Standard (*N* = 669)** | **Active (*N* = 669)** | **Placebo (*N* = 688)** |
| **Use of hypoglycemic agents** | | | | |
| Other sulfonylurea, n (%) | 472(68.6%) | 616(92.1%) | 532(79.5%) | 556(80.8%) |
| Metformin, n (%) | 651（94.6%) | 570(85.2%） | 596（89.1%） | 625（90.8%) |
| Insulin, n (%) | 12（1.7%） | 13（1.9%） | 14（2.1%) | 11(1.6%） |
| Other antidiabetic agents, n (%) | 48(7.0%) | 56(8.4%) | 46(6.9%) | 58(8.4%） |
| **Use of antihypertensive agents** | | | | |
| Perindopril, n (%) | 491(71.4%） | 471（70.4%) | 450(67.3%) | 512(74.4%) |
| Other ACE-I, n (%) | 169(24.6%) | 162(24.2%) | 163(24.4%) | 168(24.4%) |
| ARB, n (%) | 60（8.7%) | 70(10.5%) | 66(9.9%) | 64(9.3%) |
| B-blockers, n (%) | 194(28.2%) | 185(27.7%) | 175(26.2%） | 204（29.7%） |
| Diuretics, n (%) | 126（18.3%） | 141（21.1%) | 132(19.7%) | 135(19.6%) |
| Calcium antagonists, n (%) | 399（58.0%） | 395（59.0%） | 380（56.8%） | 414（60.2%） |
| Other BP lowering drug, n (%) | 212（30.8%） | 233（34.8%） | 220（32.9%） | 225（32.7% |
| **Use of lipid-lowering and antiplatelet agents** | | | | |
| Lipid-lowering agents(statins) | 157(22.8%) | 172(25.7%） | 169（25.3%） | 160（23.3%） |
| Antiplatelet agents (aspirin), n (%) | 511(74.3%) | 456(68.2%) | 488(72.9%) | 479(69.6%) |

∗All *P*-values > 0.05 between the group, data were not shown in the table

**Supplementary table3. Effect of RASGRP1 rs56254815 gene variation on primary and secondary endpoint events in patients treated with hypoglycemic therapy**

| Subgroup | Intensive, n (%) | | HR^*^ (95% CI),  *P*-value | Standard, n (%) | | HR^*^ (95% CI),  *P-*value |
| --- | --- | --- | --- | --- | --- | --- |
|  | AA (447) | AG /GG (241) |  | AA (401) | AG/GG (268) |  |
| **Primary clinical outcomes** | | | | | | |
| Combined major macro- and micro-vascular event | 57(12.8) | 34(14.1) | 0.96(0.61-1.51), 0.85 | 66(16.5) | 46(17.2) | 1.08(0.72-1.62), 0.72 |
| Major macrovascular event | 31(6.9) | 19(7.9) | 1.03(0.56-1.88), 0.92 | 31(7.7) | 18(6.7) | 0.87(0.46-1.65), 0.67 |
| MI | 3(0.7) | 1(0.4) | NA | 5(1.2) | 1(0.4) | 0.28(0.02-3.98), 0.35 |
| Stroke | 15(3.4) | 11(4.6) | 1.09(0.48-2.48), 0.84 | 20(5.0) | 12(4.5) | 0.89(0.40-1.98), 0.77 |
| Death from Cardio-cerebral vascular cause | 19(4.3) | 10(4.1) | 0.82(0.36-1.91), 0.65 | 16(4.0) | 12(4.5) | 1.28(0.56-2.92), 0.55 |
| Major microvascular event | 34(7.6) | 15(6.2) | 0.65(0.33-1.29), 0.22 | 44(11.0) | 33(12.3) | 1.21(0.74-1.98), 0.44 |
| New or worsening nephropathy | 24(5.4) | 9(3.7) | 1.64(0.69-3.90), 0.26 | 34(8.5) | 26(9.7) | 1.22(0.70-2.12), 0.49 |
| New or worsening retinopathy | 18(4.0) | 9(3.7) | 1.55(0.61-3.92), 0.36 | 18(4.5) | 12(4.5) | 1.13(0.51-2.50), 0.77 |
| **Secondary clinical outcomes** | | | | | | |
| Death from any cause | 36(8.1) | 17(7.1) | 0.86(0.46-1.59), 0.62 | 29(7.2) | 20(7.5) | 1.20(0.65-2.21), 0.57 |
| All coronary event | 29(6.5) | 10(4.2) | 0.63(0.30-1.31), 0.21 | 27(6.7) | 21(7.8) | 1.11(0.60-2.05), 0.73 |
| Major coronary | 10(2.2) | 6(2.5) | 1.05(0.35-3.17), 0.94 | 13(3.2) | 5(1.9) | 0.44(0.14-1.44), 0.18 |
| All cerebrovascular event | 25(5.6) | 11(4.6) | 0.71(0.33-1.50), 0.36 | 14(3.5) | 13(4.9) | 1.58(0.67-3.74), 0.30 |
| Major cerebrovascular event | 17(3.8) | 13(5.4) | 1.19(0.56-2.54), 0.65 | 20(5.0) | 13(4.9) | 1.00(0.46-2.18), 0.99 |
| Heart disease | 38(8.5) | 15(6.2) | 0.73(0.40-1.34), 0.31 | 46(11.5) | 25(9.3) | 0.77(0.46-1.29), 0.32 |
| All cardio-cerebral vascular event | 69(15.4) | 35(14.5) | 0.90(0.59-1.38), 0.63 | 65(16.2) | 43(16.0) | 1.03(0.68-1.55), 0.89 |
| Visual deterioration | 95(21.3) | 57(23.7) | 0.94(0.67-1.32), 0.72 | 68(17.0) | 43(16.0) | 0.93(0.62-1.38), 0.71 |
| **Continued to Supplementary table3.** Effect of RASGRP1 rs56254815 gene variation on primary and secondary endpoint events in patients treated with hypoglycemic therapy | | | | | | |
| Subgroup | Intensive, n (%) | | HR^*^ (95% CI),  *P*-value | Standard, n (%) | | HR^*^ (95% CI),  *P*-value |
|  | AA (447) | AG /GG (241) |  | AA (401) | AG/GG (268) |  |
| New or worsening microalbuminuria | 25(5.6) | 11(4.6) | 1.52(0.70-3.26), 0.29 | 31(7.7) | 10(3.7) | **0.48(0.23-1.01),0.05** |
| Combined all macro- and micro-vascular event | 182(40.7) | 103(42.7) | 0.97(0.75-1.25), 0.81 | 155(38.7) | 100(37.3) | 0.96(0.74-1.24), 0.74 |
| All microvascular events | 142(31.8) | 75(31.1) | 0.89(0.66-1.19), 0.42 | 121(30.2) | 78(29.1) | 0.97(0.72-1.31), 0.84 |
| Neuropathy | 16(3.6) | 9(3.7) | 0.90(0.37-2.18), 0.82 | 11(2.7) | 9(3.4) | 1.64(0.64-4.21), 0.31 |
| Hypoglycemia | 142(31.8) | 92(38.2) | 1.08(0.82-1.42), 0.59 | 91(22.7) | 62(23.1) | 1.00(0.72-1.42), 0.98 |
| Hospitalization | 63(14.1) | 37(15.4) | 1.01(0.67-1.56), 0.96 | 55(13.7) | 46(17.2) | 1.27(0.84-1.93), 0.27 |

^*^ All HR values are calculated with reference to the left object

**Supplementary table4. Effects of RASGRP1 rs7403531 gene variation on primary and secondary endpoint events in patients treated with hypoglycemic therapy**

| Subgroup | Intensive, n (%) | | HR^*^ (95% CI),  *P*-value | Standard, n (%) | | HR^*^ (95% CI),  *P*-value |
| --- | --- | --- | --- | --- | --- | --- |
|  | CC (250) | CT/TT (438) |  | CC (267) | CT/TT (402) |  |
| **Primary clinical outcomes** | | | | | | |
| Combined major macro- and micro-vascular event | 32(12.8) | 59(13.5) | 0.92(0.58-1.45), 0.71 | 53(19.9) | 59(14.7) | 0.71(0.47-1.06), 0.09 |
| Major macrovascular event | 18(7.2) | 32(7.3) | 0.90(0.49-1.64), 0.73 | 24(9.0) | 25(6.2) | 0.70(0.38-1.29), 0.25 |
| MI | 1(0.4) | 3(0.7) | NA | 5(1.9) | 1(0.2) | NA |
| Stroke | 13(5.2) | 13(3.0) | 0.49(0.22-1.11), 0.09 | 15(5.6) | 17(4.2) | 0.71(0.33-1.52), 0.37 |
| Death from Cardio-cerebral vascular cause | 9(3.6) | 20(4.6) | 0.97(0.42-2.23), 0.94 | 14(5.2) | 14(3.5) | 0.65(0.29-1.46), 0.29 |
| Major microvascular event | 19(7.6) | 30(6.8) | 0.72(0.38-1.34), 0.30 | 36(13.5) | 41(10.2) | 0.68(0.42-1.10), 0.12 |
| **Continued to Supplementary table 4.** Effects of RASGRP1 rs7403531 gene variation on primary and secondary endpoint events in patients treated with hypoglycemic therapy | | | | | | |
| Subgroup | Intensive, n (%) | | HR^*^ (95% CI),  *P*-value | Standard, n (%) | | HR^*^ (95% CI),  *P*-value |
|  | CC (250) | CT/TT (438) |  | CC (267) | CT/TT (402) |  |
| New or worsening nephropathy | 12(4.8) | 21(4.8) | 0.72(0.33-1.57), 0.41 | 25(9.4) | 35(8.7) | 0.75(0.43-1.31),0.31 |
| New or worsening retinopathy | 15(6.0) | 12(2.7) | **0.44(0.20-0.94), 0.03** | 15(5.6) | 15(3.7) | 0.72(0.33-1.60),0.42 |
| **Secondary clinical outcomes** | | | | | | |
| Death from any cause | 19(7.6) | 34(7.8) | 1.06(0.58-1.94), 0.86 | 25(9.4) | 24(6.0) | 0.69(0.38-1.27), 0.24 |
| All coronary event | 11(4.4) | 28(6.4) | 1.31(0.64-2.68), 0.46 | 21(7.9) | 27(6.7) | 0.79(0.43-1.44), 0.44 |
| Major coronary | 4(1.6) | 16(3.7) | 1.40(0.43-4.59), 0.58 | 9(3.4) | 9(2.2) | 0.72(0.26-1.97), 0.52 |
| All cerebrovascular event | 17(6.8) | 19(4.3) | 0.60(0.30-1.20), 0.15 | 15(5.6) | 12(3.0) | 0.51(0.22-1.21), 0.13 |
| Major cerebrovascular event | 14(5.6) | 16(3.7) | 0.58(0.27-1.22), 0.15 | 15(5.6) | 18(4.5) | 0.75(0.35-1.61), 0.46 |
| Heart disease | 17(6.8) | 36(8.2) | 1.21(0.66-2.21), 0.54 | 32(12.0) | 39(9.7) | 0.75(0.46-1.23), 0.26 |
| All cardio-cerebral vascular event | 36(14.4) | 68(15.5) | 1.06(0.69-1.61), 0.80 | 50(18.7) | 58(14.4) | 0.79(0.53-1.19), 0.26 |
| Visual deterioration | 47(18.8) | 105(24.0) | **1.41(1.00-2.00), 0.05** | 47(17.6) | 64(15.9) | 0.96(0.64-1.43), 0.84 |
| New or worsening microalbuminuria | 10(4.0) | 26(5.9) | 1.82(0.84-3.96), 0.13 | 20(7.5) | 21(5.2) | **0.48(0.25-0.92), 0.03** |
| Combined all macro- and micro-vascular event | 93(37.2) | 192(43.8) | **1.32(1.03-1.71), 0.03** | 116(43.4) | 139(34.6) | **0.71(0.55-0.93), 0.01** |
| All microvascular events | 70(28.0) | 147(33.6) | **1.35(1.01-1.81), 0.04** | 89(33.3) | 110(27.4) | **0.73(0.54-0.98), 0.04** |
| Neuropathy | 8(3.2) | 17(3.9) | 1.43(0.58-3.53), 0.44 | 9(3.4) | 11(2.7) | 0.80(0.32-2.03), 0.64 |
| Hypoglycemia | 86(34.4) | 148(33.8) | 0.95(0.72-1.25), 0.71 | 64(24.0) | 89(22.1) | 0.97(0.69-1.37), 0.87 |
| Hospitalization | 35(14.0) | 65(14.8) | 1.01(0.66-1.56), 0.95 | 50(18.7) | 51(12.7) | 0.76(0.50-1.15), 0.19 |

^*^ All HR values are calculated with reference to the left object

**Supplementary table 5. Effect of RASGRP1 rs12593201 gene variation on primary and secondary endpoint events in patients treated with hypoglycemic therapy**

| Subgroup | Intensive, n (%) | | HR^*^ (95% CI),  *P*-value | Standard, n (%) | | HR^*^ (95% CI),  *P*-value |
| --- | --- | --- | --- | --- | --- | --- |
|  | GG (237) | GA/AA (451) |  | GG (253) | GA/AA (416) |  |
| **Primary clinical outcomes** | | | | | | |
| Combined major macro- and micro-vascular event | 32(13.5) | 59(13.1) | 0.86(0.54-1.36), 0.52 | 43(17.0) | 69(16.6) | 1.02(0.67-1.55), 0.91 |
| Major macrovascular event | 18(7.6) | 32(7.1) | 0.80(0.43-1.47), 0.47 | 19(7.5) | 30(7.2) | 0.98(0.51-1.87), 0.95 |
| MI | 1(0.4) | 3(0.7) | NA | 2(0.8) | 4(1.0) | 0.57(0.07-4.61), 0.60 |
| Stroke | 11(4.6) | 15(3.3) | 0.64(0.28-1.45), 0.28 | 13(5.1) | 19(4.6) | 0.85(0.39-1.89), 0.70 |
| Death from Cardio-cerebral vascular cause | 9(3.8) | 20(4.4) | 0.89(0.38-2.08), 0.79 | 12(4.7) | 16(3.8) | 0.77(0.33-1.79), 0.54 |
| Major microvascular event | 18(7.6) | 31(6.9) | 0.75(0.40-1.42), 0.38 | 29(11.5) | 48(11.5) | 0.99(0.60-1.64), 0.97 |
| New or worsening nephropathy | 11(4.6) | 22(4.9) | 0.81(0.37-1.78), 0.60 | 21(8.3) | 39(9.4) | 0.98(0.56-1.73), 0.95 |
| New or worsening retinopathy | 14(5.9) | 13(2.9) | **0.39(0.17-0.88), 0.03** | 12(4.7) | 18(4.3) | 1.12(0.49-2.57), 0.79 |
| **Secondary clinical outcomes** | | | | | | |
| Death from any cause | 17(7.2) | 36(8.0) | 1.18(0.63-2.21), 0.61 | 22(8.7) | 27(6.5) | 0.83(0.45-1.56), 0.57 |
| All coronary event | 12(5.1) | 27(6.0) | 1.05(0.52-2.11), 0.89 | 19(7.5) | 29(7.0) | 0.77(0.41-1.44), 0.42 |
| Major coronary | 4(1.7) | 12(2.7) | 1.23(0.37-4.12), 0.74 | 6(2.4) | 12(2.9) | 1.23(0.40-3.79), 0.72 |
| All cerebrovascular event | 16(6.8) | 20(4.4) | 0.61(0.31-1.23), 0.16 | 13(5.1) | 14(3.4) | 0.68(0.28-1.64), 0.39 |
| Major cerebrovascular event | 12(5.1) | 18(4.0) | 0.71(0.33-1.53), 0.38 | 13(5.1) | 20(4.8) | 0.92(0.42-2.00), 0.83 |
| Heart disease | 18(7.6) | 35(7.8) | 1.00(0.55-1.81), 0.99 | 29(11.5) | 42(10.1) | 0.80(0.49-1.32), 0.39 |
| All cardio-cerebral vascular event | 35(14.8) | 69(15.3) | 1.01(0.66-1.54), 0.98 | 44(17.4) | 64(15.4) | 0.91(0.60-1.38), 0.66 |
| **Continued to Supplementary table 5.** Effect of RASGRP1 rs12593201 gene variation on primary and secondary endpoint events in patients treated with hypoglycemic therapy | | | | | | |
| Subgroup | Intensive, n (%) | | HR^*^ (95% CI),  *P*-value | Standard, n (%) | | HR^*^ (95% CI),  *P*-value |
|  | GG (237) | GA/AA (451) |  | GG (253) | GA/AA (416) |  |
| Visual deterioration | 47(19.8) | 105(23.3) | 1.25(0.88-1.77), 0.22 | 43(17.0) | 68(16.3) | 1.04(0.69-1.56), 0.86 |
| New or worsening microalbuminuria | 12(5.1) | 24(5.3) | 1.25(0.60-2.62), 0.55 | 19(7.5) | 22(5.3) | **0.50(0.26-0.97), 0.04** |
| Combined all macro- and micro-vascular event | 92(38.8) | 193(42.8) | 1.17(090-1.51), 0.24 | 104(41.1) | 151(36.3) | 0.84(0.65-1.10), 0.21 |
| All microvascular events | 69(29.1) | 148(32.8) | 1.19(0.89-1.60), 0.25 | 79(31.2) | 120(28.8) | 0.89(0.66-1.21), 0.47 |
| Neuropathy | 7(3.0) | 18(4.0) | 1.60(0.62-4.13), 0.33 | 8(3.2) | 12(2.9) | 0.91(0.35-2.34), 0.84 |
| Hypoglycemia | 84(35.4) | 150(33.3) | 0.95(0.72-1.26), 0.73 | 61(24.1) | 92(22.1) | 0.92(0.66-1.30), 0.65 |
| Hospitalization | 33(13.9) | 67(14.9) | 1.09(0.71-1.68), 0.70 | 45(17.8) | 56(13.5) | 0.83(0.54-1.26), 0.38 |

^*^ All HR values are calculated with reference to the left object

**Supplementary table 6. Effects of genetic variation of RASGRP1 rs7403531 on primary and secondary endpoint events in patients treated with antihypertensive therapy**

| Subgroup | Perindopril+Indapamide,n(%) | | HR^*^ (95% CI),  *P*-value | Placebo, n (%) | | HR^*^ (95% CI),  *P*-value |
| --- | --- | --- | --- | --- | --- | --- |
|  | CC (251) | CT/TT (418) |  | CC (266) | CT/TT (422) |  |
| **Primary clinical outcomes** | | | | | | |
| Combined major macro- and micro-vascular event | 38(15.1) | 56(13.4) | 0.82(0.52-1.28), 0.38 | 47(17.7) | 62(14.7) | 0.77(0.51-1.15), 0.20 |
| Major macrovascular event | 20(8.0) | 28(6.7) | 0.82(0.42-1.57), 0.54 | 22(8.3) | 29(6.9) | 0.84(0.47-1.50), 0.56 |
| MI | 3(1.2) | 4(1.0) | 0.33(0.04-2.81), 0.31 | 3(1.1) | 0(0) | NA |
| Stroke | 9(3.6) | 14(3.3) | 1.00(0.72-1.39), 0.99 | 19(7.1) | 16(3.8) | **0.48(0.24-0.97), 0.04** |
| Death from Cardio-cerebral vascular cause | 13(5.2) | 18(4.4) | 0.83(0.35-1.95), 0.67 | 10(3.8) | 16(3.8) | 1.21(0.52-2.84), 0.66 |
| Major microvascular event | 23(9.2) | 33(7.9) | 0.75(0.42-1.32), 0.31 | 32(12.0) | 38(9.0) | 0.65(0.39-1.09), 0.10 |
| New or worsening nephropathy | 14(5.6) | 26(6.2) | 0.87(0.43-1.76), 0.70 | 23(8.6) | 30(7.1) | **0.60(0.34-1.07), 0.08** |
| New or worsening retinopathy | 13(5.2) | 16(3.8) | 0.81(0.36-1.81), 0.60 | 17(6.4) | 11(2.6) | **0.33(0.15-0.73), 0.006** |
| **Secondary clinical outcomes** | | | | | | |
| Death from any cause | 21(8.4) | 30(7.2) | 0.82(0.44-1.52), 0.53 | 23(8.6) | 28(6.6) | 1.04(0.56-1.91), 0.91 |
| All coronary event | 22(8.8) | 26(6.2) | 0.70(0.38-1.26), 0.23 | 10(3.8) | 29(6.9) | **2.09(1.00-4.38), 0.05** |
| Major coronary | 9(3.6) | 12(2.9) | 0.91(0.34-2.46), 0.86 | 4(1.5) | 9(2.1) | 1.74(0.50-6.10), 0.39 |
| All cerebrovascular event | 11(4.4) | 16(3.8) | 0.78(0.32-1.90), 0.58 | 21(7.9) | 15(3.6) | **0.44(0.22-0.89), 0.02** |
| Major cerebrovascular event | 9(3.6) | 15(3.6) | 1.01(0.39-2.61), 0.98 | 20(7.5) | 19(4.5) | 0.58(0.30-1.13), 0.11 |
| Heart disease | 29(11.6) | 37(8.9) | 0.79(0.48-1.31), 0.36 | 20(7.5) | 38(9.0) | 1.18(0.67-2.09), 0.57 |
| All cardio-cerebral vascular event | 42(16.7) | 59(14.1) | 0.90(0.59-1.38), 0.64 | 44(16.5) | 67(15.9) | 1.02(0.68-1.53), 0.94 |
| **Continued to Supplementary table 6.** Effects of genetic variation of RASGRP1 rs7403531 on primary and secondary endpoint events in patients treated with antihypertensive therapy | | | | | | |
| Subgroup | Perindopril+Indapamide,n(%) | | HR^*^ (95% CI),  *P-*value | Placebo, n (%) | | HR^*^ (95% CI),  *P*-value |
|  | CC (251) | CT/TT (418) |  | CC (266) | CT/TT (422) |  |
| Visual deterioration | 43(17.1) | 74(17.7) | 1.00(0.68-1.48), 0.99 | 51(19.2) | 95(22.5) | 1.34(0.94-1.90), 0.11 |
| New or worsening microalbuminuria | 12(4.8) | 24(5.7) | 1.65(0.75-3.61), 0.21 | 18(6.8) | 23(5.5) | 0.71(0.38-1.34), 0.29 |
| Combined all macro- and micro-vascular event | 97(38.6) | 152(36.4) | 0.97(0.74-1.26), 0.79 | 112(42.1) | 179(42.4) | 1.03(0.81-1.32), 0.80 |
| All microvascular events | 70(27.9) | 116(27.8) | 1.00(0.73-1.36), 0.98 | 89(33.5) | 141(33.4) | 1.02(0.78-1.35), 0.87 |
| Neuropathy | 11(4.4) | 14(3.3) | 0.77(0.33-1.79), 0.55 | 6(2.3) | 14(3.3) | 1.55(0.55-4.37), 0.41 |
| Hypoglycemia | 77(30.7) | 125(29.9) | 1.03(0.77-1.39), 0.84 | 73(27.4) | 112(26.5) | 0.94(0.69-1.28), 0.68 |
| Hospitalization | 45(17.9) | 55(13.2) | 0.81(0.53-1.22), 0.31 | 40(15.0) | 61(14.5) | 0.94(0.62-1.42), 0.76 |

^*^ All HR values are calculated with reference to the left object

**Supplementary table7. Effects of genetic variation of RASGRP1 rs56254815 on primary and secondary endpoint events in patients undergoing antihypertensive therapy**

| Subgroup | Perindopril+Indapamide,n(%) | | HR^*^ (95% CI),  *P*-value | Placebo, n (%) | | HR^*^ (95% CI),  *P*-value |
| --- | --- | --- | --- | --- | --- | --- |
|  | AA (423) | AG/GG (246) |  | AA (425) | AG/GG (263) |  |
| **Primary clinical outcomes** | | | | | | |
| Combined major macro- and micro-vascular event | 53(12.5) | 41(16.7) | 1.31(0.84-2.03), 0.24 | 70(16.5) | 39(14.8) | 0.84(0.56-1.28), 0.42 |
| Major macrovascular event | 31(7.3) | 17(6.9) | 0.98(0.51-1.89), 0.95 | 31(7.3) | 20(7.6) | 0.87(0.48-1.57), 0.64 |
| MI | 6(1.4) | 1(0.4) | NA | 2(0.5) | 1(0.4) | NA |
| Stroke | 14(3.3) | 9(3.7) | 1.13(0.42-3.01), 0.81 | 21(4.9) | 14(5.3) | 0.85(0.40-1.78), 0.66 |
| Death from Cardio-cerebral vascular cause | 17(4.0) | 14(5.7) | 1.85(0.83-4.11), 0.13 | 18(4.2) | 8(3.0) | 0.55(0.22-1.35), 0.19 |
| Major microvascular event | 31(7.3) | 25(10.2) | 1.33(0.75-2.36), 0.32 | 47(11.1) | 23(8.7) | 0.76(0.44-1.31), 0.33 |
| New or worsening nephropathy | 23(5.4) | 17(6.9) | 1.22(0.61-2.46), 0.57 | 35(8.2) | 18(6.8) | 0.72(0.39-1.34), 0.30 |
| New or worsening retinopathy | 16(3.8) | 13(5.3) | 1.33(0.58-3.06), 0.51 | 20(4.7) | 8(3.0) | 0.86(0.35-2.10), 0.75 |
| **Secondary clinical outcomes** | | | | | | |
| Death from any cause | 31(7.3) | 20(8.1) | 1.15(0.63-2.09), 0.66 | 34(8) | 17(6.5) | 0.71(0.38-1.33), 0.28 |
| All coronary event | 29(6.8) | 19(7.7) | 1.17(0.64-2.15), 0.61 | 27(6.4) | 12(4.6) | 0.51(0.24-1.08), 0.08 |
| Major coronary | 13(3.1) | 8(3.3) | 1.26(0.47-3.34), 0.64 | 10(2.4) | 3(1.1) | 0.41(0.11-1.55), 0.19 |
| All cerebrovascular event | 17(4.0) | 10(4.1) | 0.85(0.34-2.14), 0.73 | 22(5.2) | 14(5.3) | 0.84(0.4-1.75), 0.63 |
| Major cerebrovascular event | 14(3.3) | 10(4.1) | 1.26(0.49-3.23), 0.63 | 23(5.4) | 16(6.1) | 0.92(0.46-1.83), 0.82 |
| Heart disease | 43(10.2) | 23(9.3) | 0.87(0.52-1.48), 0.61 | 41(9.6) | 17(6.5) | 0.56(0.31-1.02), 0.06 |
| All cardio-cerebral vascular event | 63(14.9) | 38(15.4) | 1.02(0.66-1.56), 0.94 | 71(16.7) | 40(15.2) | 0.79(0.52-1.19), 0.26 |
| Visual deterioration | 73(17.3) | 44(17.9) | 0.98(0.67-1.44), 0.91 | 90(21.2) | 56(21.3) | 1.02(0.73-1.44), 0.90 |
| **Continued to Supplementary table 7.** Effects of genetic variation of RASGRP1 rs56254815 on primary and secondary endpoint events in patients undergoing antihypertensive therapy | | | | | | |
| Subgroup | Perindopril+Indapamide,n(%) | | HR^*^ (95% CI),  *P*-value | Placebo, n (%) | | HR^*^ (95% CI),  *P*-value |
|  | AA (423) | AG/GG (246) |  | AA (425) | AG/GG (263) |  |
| New or worsening microalbuminuria | 28(6.6) | 8(3.3) | **0.35(0.14-0.85), 0.02** | 28(6.6) | 13(4.9) | 0.72(0.37-1.41), 0.34 |
| Combined all macro- and micro-vascular event | 155(36.6) | 94(38.2) | 1.01(0.77-1.31), 0.96 | 182(42.8) | 109(41.4) | 0.91(0.71-1.16), 0.46 |
| All microvascular events | 117(27.7) | 69(28.0) | 0.98(0.72-1.34), 0.91 | 146(34.4) | 84(31.9) | 0.88(0.67-1.16), 0.37 |
| Neuropathy | 15(3.5) | 10(4.1) | 1.10(0.47-2.57), 0.83 | 12(2.8) | 8(3.0) | 0.85(0.32-2.21), 0.37 |
| Hypoglycemia | 127(30.0) | 75(30.5) | 1.02(0.75-1.37), 0.93 | 106(24.9) | 79(30.0) | 1.04(0.77-1.40), 0.81 |
| Hospitalization | 62(14.7) | 38(15.4) | 0.98(0.64-1.50), 0.92 | 56(13.2) | 45(17.1) | 1.16(0.77-1.75), 0.47 |

^*^ All HR values are calculated with reference to the left object

**Supplementary table 8. Effects of genetic variation of RASGRP1 rs12593201 on primary and secondary endpoint events in patients in the hypotensive model**

| Subgroup | Perindopril+Indapamide,n(%) | | HR^*^ (95% CI),  *P*-value | Placebo, n (%) | | HR^*^ (95% CI),  *P*-value |
| --- | --- | --- | --- | --- | --- | --- |
|  | GG (235) | GA/AA (434) |  | GG (255) | GA/AA (433) |  |
| **Primary clinical outcomes** | | | | | | |
| Combined major macro- and micro-vascular event | 32(13.6) | 62(14.3) | 1.08(0.68-1.71), 0.75 | 43(16.9) | 66(15.2) | 0.77(0.51-1.16), 0.21 |
| Major macrovascular event | 17(7.2) | 31(7.1) | 1.07(0.54-2.11), 0.84 | 20(7.8) | 31(7.2) | 0.89(0.49-1.60), 0.69 |
| MI | 1(0.4) | 6(1.4) | 3.04(0.20-45.57), 0.42 | 2(0.8) | 1(0.2) | NA |
| Stroke | 7(3.0) | 16(3.7) | 1.73(0.59-5.02), 0.32 | 17(6.7) | 18(4.2) | 0.58(0.29-1.16), 0.12 |
| Death from Cardio-cerebral vascular cause | 12(5.1) | 19(4.4) | 0.94(0.40-2.22), 0.88 | 9(3.5) | 17(3.9) | 1.20(0.51-2.84), 0.67 |
| Major microvascular event | 17(7.2) | 39(9.0) | 1.19(0.65-2.18), 0.58 | 30(11.8) | 40(9.2) | 0.64(0.39-1.07), 0.09 |
| **Continued to Supplementary table 8.** Effects of genetic variation of RASGRP1 rs12593201 on primary and secondary endpoint events in patients in the hypotensive model | | | | | | |
| Subgroup | Perindopril+Indapamide,n(%) | | HR^*^ (95% CI),  *P*-value | Placebo, n (%) | | HR^*^ (95% CI),  *P*-value |
|  | GG (235) | GA/AA (434) |  | GG (255) | GA/AA (433) |  |
| New or worsening nephropathy | 10(4.3) | 30(6.9) | 1.50(0.70-3.20), 0.29 | 22(8.6) | 31(7.9) | **0.57(0.32-1.03),0.06** |
| New or worsening retinopathy | 10(4.3) | 19(4.4) | 1.14(0.48-2.70), 0.76 | 16(6.3) | 12(2.8) | **0.38(0.17-0.86),0.02** |
| **Secondary clinical outcomes** | | | | | | |
| Death from any cause | 18(7.7) | 33(7.6) | 1.01(0.54-1.90), 0.98 | 21(8.2) | 30(6.9) | 0.94(0.52-1.69), 0.82 |
| All coronary event | 21(8.9) | 27(6.2) | 0.67(0.37-1.22), 0.19 | 10(3.9) | 29(6.7) | 1.57(0.74-3.31), 0.24 |
| Major coronary | 7(3.0) | 14(3.2) | 1.42(0.50-4.03), 0.52 | 3(1.2) | 10(2.3) | 2.27(0.59-8.75), 0.24 |
| All cerebrovascular event | 10(4.3) | 17(3.9) | 0.81(0.32-2.03), 0.65 | 19(7.5) | 17(3.9) | **0.52(0.26-1.03), 0.06** |
| Major cerebrovascular event | 7(3.0) | 17(3.9) | 1.76(0.61-5.06), 0.29 | 18(7.1) | 21(5.5) | 0.67(0.35-1.30), 0.24 |
| Heart disease | 27(11.5) | 39(9,0) | 0.80(0.48-1.33), 0.38 | 20(7.8) | 38(8.8) | 1.09(0.62-1.92), 0.78 |
| All cardio-cerebral vascular event | 37(15.7) | 64(14.7) | 1.03(0.66-1.59), 0.91 | 42(16.5) | 69(15.9) | 0.97(0.65-1.46), 0.89 |
| Visual deterioration | 43(18.3) | 74(17.1) | 0.91(0.62-1.33), 0.62 | 47(18.4) | 99(22.9) | **1.42(1.00-2.03), 0.05** |
| New or worsening microalbuminuria | 13(5.5) | 23(5.3) | 1.20(0.56-2.57), 0.64 | 18(7.1) | 23(5.3) | 0.67(0.36-1.26), 0.22 |
| Combined all macro- and micro-vascular event | 90(38.3) | 159(36.6) | 0.98(0.75-1.28), 0.88 | 106(41.6) | 185(42.7) | 1.04(0.81-1.34), 0.74 |
| All microvascular events | 64(27.2) | 122(28.1) | 1.04(0.76-1.42), 0.83 | 84(32.9) | 146(33.7) | 1.03(0.78-1.36), 0.84 |
| Neuropathy | 9(3.8) | 16(3.7) | 1.03(0.43-2.47), 0.95 | 6(2.4) | 14(3.2) | 1.51(0.54-4.22), 0.44 |
| Hypoglycemia | 74(31.5) | 128(29.5) | 1.02(0.76-1.38), 0.89 | 71(27.8) | 114(26.3) | 0.89(0.65-1.21), 0.44 |
| Hospitalization | 39(16.6) | 61(14.1) | 1.14(0.90-1.44), 0.29 | 39(15.3) | 62(14.3) | 0.87(0.57-1.32), 0.52 |

^*^ All HR values are calculated with reference to the left object

**Supplementary table 9. The impact of *RASGRP1* genetic variation on the prognosis of primary and secondary endpoint events in patients with different genotypes**

| Subgroup | rs56254815 n (%) | | HR^*^ (95% CI),  *P*-value | rs7403531 n (%) | | HR^*^ (95% CI),  *P*-value | rs12593201 n (%) | | HR^*^ (95% CI),  *P*-value |
| --- | --- | --- | --- | --- | --- | --- | --- | --- | --- |
|  | AA  (425) | AG/GG  (932) |  | CC  (517) | CT/TT  (840) |  | GG  (490) | GA/AA  (867) |  |
| **Primary clinical outcomes** | | | | | | | | | |
| Combined major macro- and micro-vascular event | 70(16.5) | 133(14.3) | 1.03(0.76-1.39), 0.86 | 85(16.4) | 118(14.0) | 0.56(0.33-0.96),0.03 | 75(15.3) | 128(14.8) | 1.60(0.92-2.78),0.10 |
| Major macrovascular event | 31(7.3) | 68(7.3) | 0.97(0.62-1.50), 0.88 | 42(8.1) | 57(6.8) | 0.65(0.32-1.35), 0.25 | 37(7.6) | 62(7.2) | 1.50(0.71-3.16), 0.29 |
| MI | 2(0.5) | 8(0.9) | NA | 6(1.2) | 4(0.5) | NA | 3(0.6) | 7(0.8) | NA |
| Stroke | 21(4.9) | 37(4.0) | 1.04(0.58-1.85), 0.90 | 28(5.4) | 30(3.6) | 0.36(0.13-0.97), 0.04 | 24(4.9) | 34(3.9) | 2.04(0.76-5.52), 0.16 |
| Death from Cardio-cerebral vascular cause | 18(4.2) | 39(4.2) | 1.12(0.61-2.05), 0.71 | 23(4.4) | 34(4.0) | 0.75(0.30-1.89), 0.54 | 21(4.3) | 36(4.2) | 1.30(0.50-3.42), 0.59 |
| Major microvascular event | 47(11.1) | 79(8.5) | 0.98(0.66-1.44), 0.91 | 55(10.6) | 71(8.5) | **0.41(0.21-0.80), 0.01** | **47**(9.6) | **79**(9.1) | **2.10(1.06-4.19), 0.03** |
| New or worsening nephropathy | 35(8.2) | 58(6.2) | 0.95(0.60-1.49), 0.81 | 37(7.2) | 56(6.7) | 0.68(0.39-1.10), 0.11 | 32(6.5) | 61(7.0) | 2.06(0.88-4.86), 0.10 |
| New or worsening retinopathy | 20(4.7) | 37(4.0) | 0.96(0.53-1.75), 0.90 | 30(5.8) | 27(3.2) | 0.35(0.12-1.00), 0.05 | 26(5.3) | 31(3.6) | 2.34(0.81-6.76), 0.12 |
| **Secondary clinical outcomes** | | | | | | | | | |
| Death from any cause | 34(8) | 68(7.3) | 0.98(0.62-1.53), 0.91 | 44(8.5) | 58(6.9) | 0.77(0.38-1.58), 0.48 | 39(8.0) | 63(7.3) | 1.35(0.64-2.85), 0.43 |
| All coronary event | 27(6.4) | 60(6.4) | 0.92(0.58-2.06), 0.73 | 32(6.2) | 55(6.5) | 1.22(0.55-2.69), 0.62 | 31(6.3) | 56(6.5) | 0.93(0.42-2.06), 0.86 |
| **Continued to Supplementary table 9. The impact of *RASGRP1* genetic variation on the prognosis of primary and secondary endpoint events in patients with different genotypes** | | | | | | | | | |
| Subgroup | rs56254815 n (%) | | HR^*^ (95% CI),  *P*-value | rs7403531 n (%) | | HR^*^ (95% CI),  *P*-value | rs12593201 n (%) | | HR^*^ (95% CI),  *P*-value |
|  | AA  (425) | AG/GG  (932) |  | CC  (517) | CT/TT  (840) |  | GG  (490) | GA/AA  (867) |  |
| Major coronary | 10(2.4) | 24(2.6) | 0.85(0.39-1.87), 0.69 | 13(2.5) | 21(2.5) | 0.56(0.19-1.68), 0.30 | 10(2.0) | 24(2.8) | 3.16(0.96-10.42), 0.06 |
| All cerebrovascular event | 22(5.2) | 41(4.4) | 0.96(0.54-1.68), 0.87 | 32(6.2) | 31(3.7) | **0.60(0.36-1.00), 0.05** | 29(5.9) | 34(3.9) | 1.20(0.45-3.18), 0.71 |
| Major cerebrovascular event | 23(5.4) | 40(4.3) | 1.14(0.66-1.96), 0.65 | 29(5.6) | 34(4.0) | 0.41(0.16-1.07), 0.07 | 25(5.1) | 38(4.4) | 1.99(0.75-5.29), 0.17 |
| Heart disease | 27(6.4) | 83(8.9) | 0.79(0.53-1.18), 0.24 | 49(9.5) | 75(8.9) | 1.12(0.57-2.21), 0.75 | 47(9.6) | 77(8.9) | 0.89(0.45-1.77), 0.75 |
| All cardio-cerebral vascular event | 71(16.7) | 141(15.1) | 0.98(0.73-1.32), 0.89 | 86(16.6) | 126(15) | 0.86(0.51-1.46), 0.57 | 79(16.1) | 133(15.3) | 1.20(0.70-2.04), 0.51 |
| Visual deterioration | 90(21.2) | 173(18.6) | 0.96(0.74-1.24), 0.73 | 94(18.2) | 169(20.1) | 1.20(0.74-1.95), 0.45 | 90(18.4) | 173(20.0) | 0.98(0.61-1.60), 0.95 |
| New or worsening microalbuminuria | 28(6.6) | 49(5.3) | **0.53(0.31-0.89), 0.02** | 30(5.8) | 47(5.6) | 1.77(0.68-4.59), 0.24 | 31(6.3) | 46(5.3) | 0.52(0.20-1.35), 0.18 |
| Combined all macro- and micro-vascular event | 182(42.8) | 358(38.4) | 0.93(0.77-1.12), 0.42 | 209(40.4) | 331(39.4) | 0.98(0.70-1.39), 0.91 | 196(40) | 344(39.7) | 1.05(0.74-1.48), 0.80 |
| All microvascular events | 146(34.4) | 270(29.0) | 0.88(0.71-1.08), 0.22 | 159(30.8) | 257(30.6) | 0.94(0.64-1.40), 0.77 | 148(30.2) | 168(19.4) | 1.10(0.74-1.64), 0.64 |
| Neuropathy | 12(2.8) | 33(3.5) | 1.10(0.57-2.13), 0.79 | 17(3.3) | 28(3.3) | 0.69(0.22-2.19), 0.53 | 15(3.1) | 30(3.5) | 1.75(0.54-5.72), 0.35 |
| Hypoglycemia | 106(24.9) | 281(30.2) | 1.08(0.87-1.33), 0.50 | 150(2.9) | 237(28.2) | 0.96(0.66-1.40), 0.84 | 145(29.6) | 242(27.9) | 0.95(0.66-1.38), 0.79 |
| Hospitalization | 56(13.2) | 145(15.6) | 1.17(0.87-1.58), 0.30 | 85(16.4) | 116(13.8) | 0.82(0.48-1.41), 0.47 | 78(15.9) | 123(14.2) | 1.22(0.71-2.12), 0.48 |

^*^ All HR values are calculated with reference to the left object

**Supplementary Figure 1. Linkage disequilibriumdiagram of candidate SNPs**


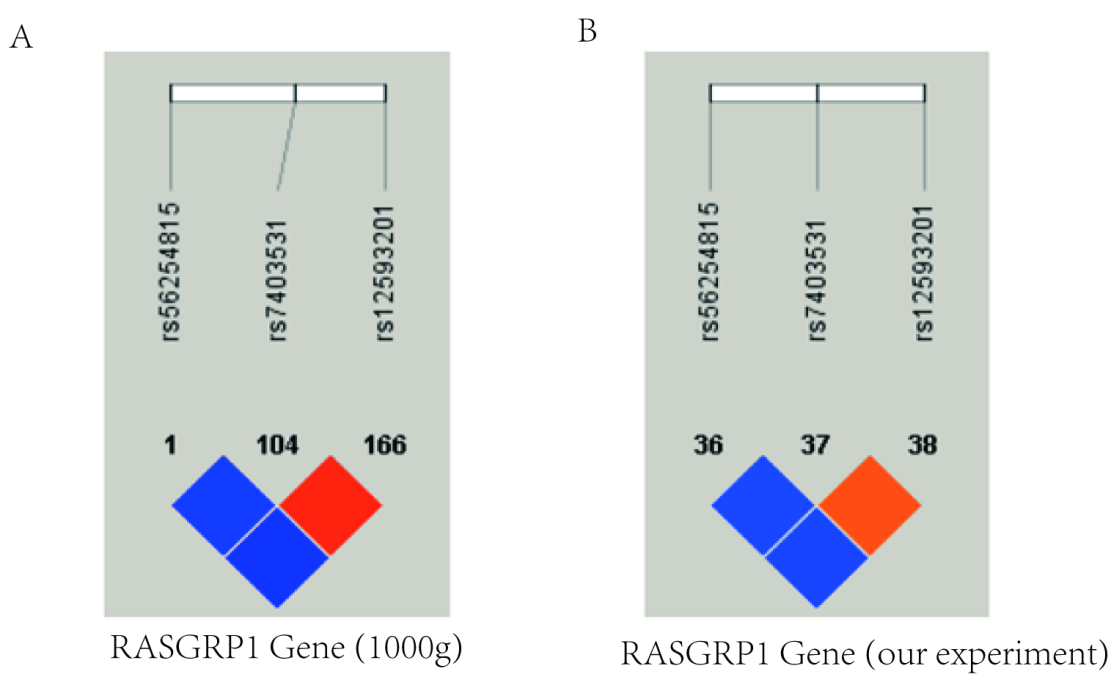

Supplement: Supplementary file 1 — Supplementary Material 1 [file 12933_2024_2267_MOESM1_ESM.docx]
