# Supplementary material for: Association of RASGRP1 polymorphism with vascular complications in Chinese diabetic patients with glycemic control and antihypertensive treatment
Source: Cardiovasc Diabetol. 2024 May 10;23:166. doi: 10.1186/s12933-024-02267-2 (PMC11088008; doi:10.1186/s12933-024-02267-2)
Supplement: Supplementary file 2 — Supplementary Material 2 [file 12933_2024_2267_MOESM2_ESM.docx]

**Association of RASGRP1 Polymorphism with Vascular Complications in Chinese diabetic patients with glycemic Control and Antihypertensive treatment**

Jiecan Zhou^1,2,3,5*^ Bo Xu^1,2,3,5^ Fazhong He^3,4^ Yan Shu^5^ Xiaoping Chen^3,4,1^ Zhaoqian Liu^3,4,1^ Bao Sun^1,3,4,5*^ Wei Zhang^4,5,1*^

**Abstract**

**Background:** Studies have shown that *RASGRP1* was potently associated with the onset of type 2 diabetes mellitus (T2DM), and *RASGRP1* rs7403531 was significantly correlated with islet function in T2DM patients. However, the effect of *RASGRP1* polymorphism on blood glucose and blood pressure in T2DM patients after continuous treatment has yet to be fully elucidated.

**Objective:** This study aimed to explore the association between *RASGRP1* genetic polymorphism and cardiovascular complications in T2DM patients, so as to provide more evidence for the individualized treatment of T2DM patients.

**Methods:** We retrospectively analyzed a large-scale multicenter drug clinical study cohort that based on a 2×2 factorial (glucose control axis and blood pressure lowering axis) randomized controlled design, with follow-up for 5 years. The major vascular endpoint events included cardiovascular death, non-fatal stroke, coronary heart disease, new-onset or worsening renal disease, and diabetic retinopathy. RASGRP1 rs12593201, rs56254815 and rs7403531 were finally selected as candidate [single](D:/%E8%BD%AF%E4%BB%B6/Software/%E6%9C%89%E9%81%93%E8%AF%8D%E5%85%B8/Dict/8.5.3.0/resultui/html/index.html#/javascript:;) [nucleotide](D:/%E8%BD%AF%E4%BB%B6/Software/%E6%9C%89%E9%81%93%E8%AF%8D%E5%85%B8/Dict/8.5.3.0/resultui/html/index.html#/javascript:;) [polymorphism](D:/%E8%BD%AF%E4%BB%B6/Software/%E6%9C%89%E9%81%93%E8%AF%8D%E5%85%B8/Dict/8.5.3.0/resultui/html/index.html#/javascript:;)s. Mixed linear model and Cox hazard ratio (HR) model were used for data analysis with IBM SPSS (version 20.0 for windows; Chicago, IL).

**Results:** Our study enrolled 1357 patients with high-risk diabetes, with a mean follow-up duration of 4.8 years. *RASGRP1* rs7403531 was associated with vascular events in hypoglycemic and antihypertensive therapy. Specifically, compared with CC carriers, patients with CT/TT genotype had fewer major microvascular events (HR=0.41, 95% confidence interval (CI) 0.21-0.80, *P*=0.009), and reduced the risk of major eye disease events (HR=0.44, 95% CI 0.20-0.94, *P*=0.03). For glucose lowering axis,CT/TT carriers had a lower risk of secondary nephropathy (HR=0.48, 95% CI 0.25-0.92, *P*=0.03) in patients with standard glycemic control. For blood pressure lowering axis, all cerebrovascular events (HR=2.24, 95% CI 1.11-4.51, *P*=0.025) and stroke events (HR=2.07, 95% CI 1.03-4.15, *P*=0.04) were increased in patients with CC genotype compared to those with CT/TT genotype in the placebo group, respectively. Furthermore, patients with CC genotype showed a reduced risk of major cerebrovascular events in antihypertensive group (HR=0.36, 95% CI 0.15-0.86, *P*=0.021). For *RASGRP1* rs56254815, compared with the AA genotype carriers, the systolic blood pressure of AG/GG carriers in the antihypertensive group decreased by 1.5mmhg on average (*P*=0.04). In the placebo group, the blood pressure of AG/GG carriers was 1.7mmHg higher than that of AA carriers (*P*=0.02).

**Conclusion:** We found that patients with G allele of *RASGRP1* (rs56254815) showed a better antihypertensive therapy efficacy in T2DM patients. The rs7403531 T allele could reduce the risk of major microvascular events and major eye diseases in T2DM patients receiving either hypoglycemic or antihypertensive therapy. Our findings suggest that *RASGRP1* genetic polymorphism might predict the cardiovascular complications in T2DM patients.

**Keywords:** RASGRP1; rs7403531; polymorphism; Vascular Complications; type 2 diabetes; individual drug treatment

1.The First Affiliated Hospital, Hunan Provincial Clinical Medical Research Center for Drug Evaluation of Major Chronic Diseases，Hengyang Medical School, University of South China, Hengyang 421001, Hunan, China

2.The First Affiliated Hospital, Hengyang Clinical Pharmacology Research Center, Hengyang Medical School, University of South China, Hengyang 421001, Hunan, China

3.Department of Clinical Pharmacology, Xiangya Hospital, Central South University, 110 Xiangya Rode, Kaifu district, Changsha, Hunan 410008, P.R. China.

4.Pharmacogenetics Research Institute, Institute of Clinical Pharmacology, Hunan Key Laboratory of Pharmacogenetics, Central South University, Changsha, Hunan 410078, China.

5.Department of Pharmaceutical Sciences, School of Pharmacy, University of Maryland, Baltimore, Maryland, USA.

***Correspondence:**

Prof. Jiecan Zhou, The First Affiliated Hospital, Hunan Provincial Clinical Medical Research Center for Drug Evaluation of Major Chronic Diseases，Hengyang Medical School, University of South China, Hengyang 421001, Hunan, China

Tel: +86-734-8279018, Fax: +86-734-82792009, E-mail: [zhoujiecan@fsyy.usc.edu.cn](mailto:zhoujiecan@fsyy.usc.edu.cn), ORCID ID: 0000-0002-3037-3997

Prof. Wei Zhang, Department of Clinical Pharmacology, Xiangya Hospital, Central South University, 110 Xiangya Rode, Kaifu district, Changsha, Hunan 410008, P.R. China. Tel: +86-731-8480-5380, Fax: +86-731-8235-4476, E-mail: csuzhangwei@csu.edu.cn

Prof.Bao Sun, Department of Clinical Pharmacology, Xiangya Hospital, Central South University, 110 Xiangya Rode, Kaifu district, Changsha, Hunan 410008, P.R. China. Email Bao Sun: scy_csu2016@csu.edu.cn

**Others authors**

Email Bo Xu: [xuboj20@foxmail.com;](mailto:xuboj20@foxmail.com;) Bo Xu ORCID ID: 0000-0003-0594-9103

Email Fazhong He: fazhong2006@ext.jnu.edu.cn

Email Yan Shu: yshu@rx.umaryland.edu

Email Xiaoping Chen: chenxp74@hotmail.com

Email Zhaoqian Liu: zqliu@csu.edu.cn

**Introduction**

Type 2 diabetes mellitus (T2DM) is a complex metabolic disease with rapidly increasing prevalence worldwide. Characteristics of T2DM include long-term elevation of blood glucose levels due to reduced insulin secretion by pancreatic islet cells and insulin resistance in peripheral tissue [1,2]. Persistent hyperglycemia can trigger a pathological cascade that leads to more serious diseases such as cardiovascular disease, renal failure, metabolic syndrome, or hormonal dysfunction [3].

In addition to well-known risk factors such as overweight, unhealthy lifestyle, metabolic changes, previous diagnosis of gestational diabetes, or family history of cardiovascular disease, genetic variations also play an important role in T2DM, with estimates ranging from 20% to 80%. So far, genome-wide association studies (GWAS) have identified at least 75 loci associated with T2DM or its traits, but there was no breakthrough in the understanding of the pathological mechanism of the disease or drug therapy [4,5]. Moreover, these genetic variations explain only 10-15% of the genetic probability of this diseases [6], and the study of genetic mechanisms is still an area of concern.

At present, there is increasing evidence that T2DM can also be defined as an auto-inflammatory disease, in which immune dysregulation disrupts insulin production in pancreatic islet beta cells and signal transduction to target tissues [7,8]. This overturns the traditional understanding of diabetes, suggesting that genes regulating immunity may influence the process of T2DM. RASGRP1 is a guanine nucleotide exchange factor (GEF) that activates RAS protein through the conversion of GDP to GTP initiate a cascade reaction of RAF/MEK/ERK signaling pathway downstream [9]. In its protein structure, RASGRP1 has an atypical pair of "EF" arms that bind to calcium ions and a diacylglycerol (DAG) binding domain (Figure 1A). Studies have shown that these two special domains are crucial for the function of RASGRP1 protein [10]. RASGRP1 is well known for regulating the immune response of T cells, B cells and other lymphocytes through the signaling cascade of RAS/RAF/MEK/ERK, influencing the progression of a large number of immune diseases such as acute leukemia (AML), solid tumors, lupus erythematosus (SLE) and their drug therapy [11]. Recent studies have shown that multiple single nucleotide polymorphisms (SNPs) in *RASGRP1* gene were related to type 2 diabetes mellitus (T2DM) [12,13], suggesting that *RASGRP1* may drive the process of T1DM through immunity, but whether it is also through a similar possible mechanism for T2DM is unclear.

In recent years, many studies have reported that *RASGRP1* was strongly associated with cardiovascular diseases and metabolic diseases [14]. Studies on European populations have found that *RASGRP1* is negatively correlated with HbA1c [15]. A number of studies have identified rs7403531, a site located in the *RASGRP1* gene intron 2, as a new susceptibility site for T2DM in Chinese populations [16,17], which has been verified in European populations derived from the DIAbetes Genetics Replication And meta-analysis (DIAGRAM) consortium data [18]. However, there is no linkage disequilibrium between this site and known susceptible sites of T1DM, suggesting that the pathogenesis of rs7403531-associated signal driving T2DM may be different from that of T1DM. *RASGRP1* expressed by cardiomyocytes mediates the phosphorylation of p38 MAPK and the expression of periostin induced by Angiotensin II (AngII) [19]. Inhibiting the expression of *RASGRP1* may contribute to the improvement in heart failure. *RASGRP1* expressed in the kidney mediates the functional inhibition and expression of sodium-chloride co-transporter (NCC) in the distal convoluted tubule (DCT) cells induced by PE through the MAPK signaling pathway [20,21]. NCC is an important protein regulating blood pressure and a major target of thiazine antihypertensive drugs. This suggests that *RASGRP1* is not only associated with the onset of T2DM, but also with the cardiovascular prognosis of T2DM treatment.

Many genetic variants that affect the susceptibility to T2DM are associated with the risk of vascular events [22], prompting us to explore the association between these reported pathogenic gene polymorphisms and vascular complications of T2DM. Previous studies in our research group have also found that *TRIB3* rs2295490, *POLR2A* rs71541942, *ATF6* rs12086247, *SMARCD3* rs58125572 and other gene polymorphisms were significantly correlated with vascular complications under different hypoglycemic or hypertensive treatment modes [23,24]. Based on previous research strategies and a large T2DM clinical trial cohort, we conducted an association study between common genetic variations of *RASGRP1* and vascular complications under hypoglycemic or hypertensive therapy, aiming to understand the genetic effects of T2DM on drug therapy from a more comprehensive perspective.

**Methods**

**Study design and patients**

We conducted a retrospective study on the cohort from Action in Diabetes and Vascular disease: preterAx and diamicroN-MR Controlled Evaluation (ADVANCE) clinical trial at 61 centers in China, with a follow-up period of 5 years. Approval to conduct the trial was obtained from the ethics committee of each study center, and all participants provided written informed consent (registration number NCT00145925). Detailed rationale, design, follow-up schedule and clinical endpoints of ADVANCE trial have been described in previous studies [25].

In brief, it was a 2 × 2 factorial randomized controlled trial. Patients with T2DM were randomly (1:1) assigned to receive perindopril-indapamide or matching placebo for blood pressure control, and modified-release gliclazide based intensive or local standard therapy for glycaemic control. For blood pressure control, participants were treated for 6 weeks as run-in period with combination of perindopril and indapamide, then randomly grouped into fixed combination regimen (perindopril/indapamide, initially 2.0/0.625 mg daily, increasing to 4.0/1.25 mg daily after 3 months) or matching placebo. For hypoglycemic control, an open label, randomized protocol was implemented to an intensive glucose control or to local standard therapy based on local guidelines. The intensive glucose control was defined as the use of gliclazide modified release-based regimen (30–120 mg daily) and other oral agents, then insulin aiming for a HbA1c value of 6.5% or lower. The local standard treatment was defined as the patients who continue with their usual glucose control regimens, which may include any therapy except the use of gliclazide.

The major vascular endpoints include death from cardio-cerebral vascular diseases, nonfatal stroke or nonfatal myocardial infraction, and new or worsening renal or diabetic eye disease. Other vascular events such as cerebrovascular events (include death due to cerebrovascular disease, stroke, transient ischemic attack, and subarachnoid hemorrhage), coronary events (include myocardial infarction, angina pectoris, myocardial ischemia, and sudden death), heart disease (coronary heart disease, heart failure, atrial fibrillation), new or worsening microalbuminuria, and visual deterioration were also evaluated both jointly and separately. In our study, there was no other pre-specified criterion for the levels of blood pressure, HbA1c or other baseline clinical characteristics at entry.

**SNP selection and genotyping**

Candidate tag SNPs of *RASGRP1* were initially selected based on literature, the database from NCBI (https://www.ncbi.nlm.nih.gov/ snp/), the 1000 Genomes Project (https://www.ncbi. nlm.nih.gov/variation/tools/1000 genomes/), and the Haploview 4.2 (Cambridge, MA, USA). Moreover, all of the selected SNPs had a minimum allele frequency > 5% in the Southern Han Chinese population. The SNPs rs12593201, rs56254815 and rs7403531 were genotyped by SNPscan™ (Genesky Biotechnologies, Shanghai, China). To evaluate the accuracy of the genotyping results, 5% of samples were genotyped in duplicate and showed 100% concordance. At last, a total of 1357 patients from 61 clinical centers were successfully genotyped.

Primer information for sequencing: rs12593201, 5F1-Seq: CATGGTAACAGGCACTACTGGTGTCTATATCC; 5F2-Seq: CATGGTAACAGGCACTACTGGTGTCTATACCT; 3F-Seq: AAGCTTTTGTTGAGTATGTTTTCATAAGTTTG. rs56254815 5F1-Seq: GGTTTCCTTCCCACTTGAGCTAGAGTGC; 5F2-Seq: GGTTTCCTTCCCACTTGAGCTAGAGTGT; 3F-Seq: AAAATGAAGGAAAATAAAGGAATGTCACC, rs7403531 5F1-Seq: CCATCTCAACTTTCCTTTATGGCTTGC; 5F2-Seq: CCATCTCAACTTTCCTTTATGGCTCGT; 3F-Seq: TAGCATCTTCCCATAGATACTCATATGTGA

**Statistical Analysis**

Continuous data were presented as mean values ± standard deviation (SD) or mean (inter-quartile range, IQR), and frequencies or percentages for categorical variables. The Hardy–Weinberg equilibrium (HWE) for the study participants was assessed with the χ2-test. Comparisons of difference in baseline characteristics among the phenotypes were performed by independent-samples t-test or Wilcoxon rank sum test, appropriately. Mixed linear model was performed in plasma glucose, lipid levels and blood pressure between *RASGRP1* genotype with adjustment for sex, age, duration of the disease, body mass index (BMI), combined medication and drug dosage. Cox proportional hazards models were used to investigate the relationship between genetic variation and the risk of vascular events, adjusting for history of vascular disease, baseline sex, age, duration of the disease, combined medication, drug dosage and clinical biochemical factors (i.e., potassium concentration and low-density lipoprotein). The cumulative risk of vascular related events was calculated as (1-hazard ratio [HR]) × 100%. Three genetic models (additive, dominant, and recessive genetic models) were used to test any differences of categorical variables and quantitative variables among the four treatment groups. A two-tailed *P*-value <0.05 was considered significant. All the analyses were performed with IBM SPSS (version 20.0 for windows; Chicago, IL).

**Results**

**Baseline Characteristics, Genotyping Results and Outline of Major Findings**

We obtained 1500 peripheral venous blood samples in patients with T2DM from ADVANCE (China center), of which 132 (8.8%) samples were rejected due to sample quality and 11 (0.7%) samples were not genotyped successfully. Since typing errors can significantly reduce the degree of linkage disequilibrium between this site and other linkage sites, we intuitively estimated the overall quality of experimental data by constructing LD map of *RASGRP1* gene (Supplement Figure 1). The overall distribution scheme of the experiment was shown in Figure 1B. A total of 1357 DNA samples were successfully genotyped for *RASGRP1* rs12593201, rs56254815, rs7403531. All of the sites were in Hardy-Weinberg equilibrium among random groups (Supplementary table 1). In hypoglycemic therapy, there was no significant difference in the clinical baseline characteristics between the standard hypoglycemic group and the intensified hypoglycemic treatment group, and there was no significant difference between the antihypertensive treatment group and the placebo group (Supplementary table 2).

**Effects of *RASGRP1* polymorphism on blood glucose and vascular complications in hypoglycemic therapy**

During the follow-up period, the mean HbA1c of patients in the intensive hypoglycemic treatment group was 6.77%, while that of the standard hypoglycemic group was 7.37%. We evaluated three gene models (dominant, recessive, additive) and used the results of the dominant model finally. For *RASGRP1* 12593201, the mean HbA1c level was 6.78% vs 6.77% in the enhanced hypoglycemic group, and 7.29% vs 7.42% in the standard glycemic control group (GG vs GA/AA). For *RASGRP1* rs56254815, the mean HbA1c level was 6.74% vs 6.82% in the enhanced hypoglycemic group, and 7.36% vs 7.37% in the standard glycemic control group (AA vs AG/GG). For *RASGRP1* rs7403531, the mean HbA1c level was 6.76% vs 6.78% in the enhanced hypoglycemic group, and 7.31% vs 7.40% in the standard glycemic control group (CC vs CT/TT). After the analysis of the mixed linear model, we found that the genetic variation of *RASGRP1* had no significant effect on the hypoglycemic effect in either the intensive hypoglycemic group or the standard hypoglycemic group (Figure 2). In addition, fasting blood glucose (FPG) had similar trend with HbA1c during the follow-up period between different genotypes in each point, but also no statistical differences (data not shown).

During follow-up period, 225 patients with T2DM developed major macrovascular and/or microvascular events. Among the three sites rs56254815 (Supplementary table 3), rs7403531 (Supplementary table 4), rs12593201 (Supplementary table 5), rs7403531 was mainly closely associated with vascular events. In all patients, there were a total of 126 microvascular events, and the number of major microvascular events was 49 (7.1%) in patients who received intensive hypoglycemic therapy. The number of major microvascular events was 77 (11.5%) in patients who received standard oral glucose-lowering therapy. Compared with the standard hypoglycemic, intensive glucose control reduced major microvascular events (HR=0.61, 95% confidence interval (CI) 0.42-0.89, *P*=0.011) (Figure 3A). The number of major microvascular events in rs7403531 CT/TT carriers was 71 (8.5%), and the number of such vascular events in CC carriers was 55 (10.6%). Major microvascular events were reduced in CT/TT genotype carriers compared with CC carriers (HR=0.41, 95% CI 0.21-0.80, *P*=0.009) (Figure 3A). This suggests that intensive glucose-lowering in type 2 diabetic patients can reduce the risk of major microvascular events, while *RASGRP1* rs7403531 CT/TT carriers are less likely to cause major microvascular events.

As can be seen from Supplementary table 6, *RASGRP1* rs7403531 was closely related to eye disease events. We observed that CT/TT carriers who received intensive hypoglycemic therapy had a reduced risk of major eye disease events (HR=0.44, 95% CI 0.20-0.94, *P*=0.03), but an increased risk of minor eye disease (HR=1.41, 95% CI 1.00-2.00, *P*=0.05) compared with rs7403531 CC carriers. At the same time, in these gene carriers, the number of secondary eye disease events increased with intensive hypoglycemic therapy compared with standard therapy (HR=1.54, 95% CI 1.12-2.12, *P*=0.008) (Figure 3B and C).

Nephropathy-related vascular events were another complication associated with rs7403531 during hypoglycemic therapy. Among all T2DM patients, 33 (4.8%) patients in the intensive hypoglycemic group had major nephropathy events, while 60 (9.0%) patients in the standard group had major nephropathy events. Compared with standard hypoglycemic, intensive hypoglycemic reduced major kidney disease events (HR=0.55, 95% CI 0.35-0.85, *P*=0.007). and in all the T2DM patients, the number of major renal events in CC carriers was 37 (7.2%), while the number was 56 (6.7%) in CT/TT genotype patients, there were no significant differences between the two genotypes carriers (HR=0.66, 95% CI 0.39-1.10, *P*=0.11). CT/TT carriers had a lower risk of secondary nephropathy in the standard glucose-lowering group (HR=0.48, 95% CI 0.25-0.92, *P*=0.03) (Figures 3D and E).

Combined with all microvascular events, we found that rs7403531 CT/TT genotype carriers in the intensive hypoglycemic group increased the risk of all microvascular events (HR=1.35, 95% CI 1.01-1.81, *P*=0.04). And for CT/TT genotype carriers, intensive hypoglycemic control increased the risk of all microvascular events (HR=1.30, 95% CI 1.00-1.68, *P*=0.04). In the standard glucose-lowering group, the CT/TT genotype carriers had a reduced risk of all microvascular events (HR=0.73, 95% CI 0.54-0.98, *P*=0.04). When we combined all the microvascular events and major vascular events, we found that in the intensive hypoglycemic group, CT/TT genotype carriers would also increase the risk of all macrovascular events (HR=1.32, 95% CI 1.03-1.71, *P*=0.03), and in the standard hypoglycemic group, CT/TT genotype carriers had a reduced risk of all macrovascular event (HR=0.71, 95% CI 0.55-0.93, *P*=0.01). Consistent with this, intensive hypoglycemic increased the risk of such events in CT/TT patients (HR=0.73, 95% CI 0.58-0.92, *P*=0.007) (Figure 3F and G). It was suggested that intensive hypoglycemic therapy would increase the risk of vascular events in *RASGRP1* rs7403531 CT/TT patients, while on the contrary, standard hypoglycemic therapy would benefit patients in terms of vascular complications.

**Effects of *RASGRP1* polymorphism on blood pressure and vascular complications in antihypertensive therapy**

During follow-up period, the systolic blood pressure (SBP) and diastolic blood pressure (DBP) values were 133.6mmHg and 75.2mmHg, respectively, in the T2DM patients treated with perdoperine-indapamide tablets. The mean SBP of the placebo matched group was 137.3mmHg and the mean DBP was 76.5mmHg. For *RASGRP1* rs7403531, SBP increased by an average of 2.2 mm Hg in CT + TT genotype patients compared with CC genotype carriers in the antihypertensive group, and by an average of 0.4 mmHg in the placebo group (without significant difference) (Figure 4A). For *RASGRP1* rs12593201, the SBP of GA+AA carriers increased by an average of 1.3mmHg compared with the GG carriers of *RASGRP1* (rs12593201) in the antihypertensive group (Figure 4B). Also in the placebo group, there was no difference in SBP between the two genotypes. For *RASGRP1* rs56254815, compared with AA genotype carriers of *RASGRP1* rs56254815, SBP of AG/GG carriers decreased by 1.5 mmHg on average (*P*=0.04), and DBP decreased by 1.1 mmHg on average in the antihypertensive group. In the placebo group, the blood pressure of AG/GG carriers increased by 1.7mmHg (*P*=0.02) compared with that of AA carriers (Figure 4C). There was no significant difference in DBP between genotypes for any of the three sites, either in the active antihypertensive group or the placebo group (data not shown). The above results suggested that patients with *RASGRP1* rs56254815 AG/GG genotype had better response to antihypertensive therapy.

In order to explore the effect of antihypertensive therapy on the prognosis of T2DM patients, we performed the same analysis on vascular complications in the targeted subgroup of antihypertensive therapy. It was found that rs7403531, rs56254815 and rs12593201 were significantly correlated with vascular complications after antihypertensive therapy (Supplementary table 6-9). According to the results of Supplement Figure 1, there was a linkage disequilibrium between rs7403531 and rs12593201 (D '=0.913, r^2^=0.763), and the correlation between rs12593201 and vascular events may be caused by the linkage disequilibrium between rs7403531 and rs12593201. In our detailed analysis of rs7403531, we found that in the placebo group, compared with patients with CT/TT genotype, all cerebrovascular events increased in CC genotype patients (CC vs CT/CC:HR=2.24, 95% CI 1.11-4.51, *P*=0.025). For blood pressure lowering axis, there was no significant difference in the occurrence of cerebrovascular events risk between CC and CT/TT genotypes (HR=0.78, 95% CI 0.32-1.90, P=0.58). Active antihypertensive therapy in CC patients resulted in approximately reduced risk of all cerebrovascular events. (placebo vs antihypertensive therapy: HR=0.47, 95% CI 0.21-1.03, *P*=0.06) (Figure 5A). We also found that antihypertensive therapy for CC patients reduced the risk of major cerebrovascular events (HR=0.36, 95% CI 0.15-0.86, *P*=0.021) (Figure 5B). This suggested that patients with *RASGRP1* rs7403531 CC genotype were more likely to trigger cerebrovascular events, and active antihypertensive therapy for this part of the population would reduce the risk of cerebrovascular events.

In the placebo group, patients with rs7403531 CC genotype have an increased number of stroke events compared with CT/TT genotype (HR=2.07, 95% CI 1.03-4.15, *P*=0.04), and antihypertensive therapy in these patients resulted in a reduced risk of all strokes (HR=0.35, 95% CI 0.15-0.84, *P*=0.019) (Figure 5C). However, the results of coronary heart disease events were opposite. In the placebo group, compared with CT/TT genotype patients, CC genotype patients have lower risk of coronary heart disease events (HR=0.48, 95% CI 0.23-1.00, *P*=0.049). This suggested that the rs7403531 CC genotype was inconsistent in the prognosis of cardiovascular events and cerebrovascular events, which might be related to the different mechanisms of its function in different tissues.

We also observed no significant effect of various treatments on major renal events (Figure 5E). In the placebo group, CC genotype carriers have a higher risk of developing major eye diseases compared to CT+TT genotype carriers (HR=2.39, 95% CI 1.07-5.30, *P*=0.033) (Figure 5F). For both CC genotype (HR=1.36, 95% CI 0.55-3.34, *P*=0.51) and CT/TT genotype (HR=0.55, 95% CI 0.22-1.35, *P*=0.19) patients, antihypertensive therapy did not alter the risk of major eye disease events.

**Discussion**

For patients with T2DM complicated with hypertension, comprehensive control of blood lipid, blood pressure and blood glucose level are extremely important to reduce the risk of vascular disease in diabetics [26]. In this study, a 2×2 factorial design was used to explore the effectiveness of hypoglycemic treatment and antihypertensive treatment in all enrolled patients. In our cohort, T2DM patients receiving intensive glucose therapy were treated with metformin, insulin, glucosidase inhibitors and other glucose-lowering drugs to achieve HbA1c levels below 6.5%, in addition to gliclazide sustained release tablets. However, for T2DM patients receiving standard blood glucose regimen, the final overall HbA1c level remain above 7.0% according to the Chinese guidelines for diabetes prevention and treatment and the clinical practice by using metformin, sulfonylureas (except glycolide), insulin and other conventional hypoglycemic drugs. As depicted in figure 2, in the study model of hypoglycemic therapy, the genetic variation of the three candidate sites of *RASGRP1* did not significantly impact the HbA1c level of patients in all subgroups undergoing hypoglycemic therapy. Previous studies have shown that compared with non-diabetic or normal blood glucose group, diabetic or hyperglycemia group had lower *RASGRP1* expression level, while *RASGRP1* expression level was negatively correlated with HbA1c [15]. Nonetheless, the absence of association between *RASGRP1* rs7403531 and HbA1c levels suggests that this locus is unlikely to influence the gene expression regulationr.

In our study, we examined three candidate SNPs within the *RASGRP1* gene, all of which exhibited MAF greater than 5% and were located in intron regions of *RASGRP1*. Our analysis revealed no significant association between these SNPs and HbA1c levels in each treatment mode. Notably, two of these SNPs, excluding rs56254815, demonstrated a significant association with vascular complications in T2DM patients. Furthermore, a linkage disequilibrium analysis indicated that rs12593201 and rs7403531 were in strong linkage disequilibrium, suggesting a similar impact on the risk of vascular concurrent events among carriers. rs7403531 has been proved to be correlated with T2DM in a number of correlation studies in Chinese population [16-18]. Our findings indicated that rs7403531 played an important role in the pathogenesis of T2DM or in drug treatment model. We found that *RASGRP1* rs56254815 AG/GG carriers exhibited higher SBP. Such genotype carriers can receive better curative effect after receiving antihypertensive treatment.

In the hypoglycemic analysis model, our data showed that compared with the standard hypoglycemic, intensive glucose control reduced 5.6% main nephropathy and 4.4% main microvascular events, which was far from achieving UKPD research 25% [27], indicating that intensive glucose control of T2DM patients benefit less remarkable than most people think. In the whole enrolled population, the major microvascular events in rs7403531 CT/TT genotype carriers were decreased compared with CC genotype carriers, indicating that CT/TT genotype carriers were less likely to cause major microvascular events. Intensive hypoglycemic therapy resulted in a reduced risk of major eye events in CT/TT genotype carriers, and the T allele was a beneficial gene. In the standard glucose-lowering group, the T allele also benefited patients with secondary renal disease risk. In addition,, we also observed that enhanced glucose lowering increased the risk of secondary eye diseases such as vision loss, and that enhanced glucose lowering was more likely to occur in T allele carriers. In the combined analysis of all microvascular and macrovascular events, we found that carriers of the T allele increased the risk of all major microvascular events in the intensive hypoglycemic group, while in the standard hypoglycemic group, the risk of all major microvascular events was decreased. These findings suggest that in clinical practice, appropriate hypoglycemic treatment methods should be selected according to patients' maximum benefit orientation based on comprehensive measurement. Intensive hypoglycemic treatment can benefit patients to some extent but increase the risk of alternative events. *RASGRP1* rs740353 T allele increased the risk of all vascular events overall, yet it provided advantages in terms of major microvascular event.

In the antihypertensive analysis model, genetic variation of rs7403531 did not affect the efficacy of antihypertensive therapy. In the placebo group, compared with CT/TT genotype patients, CC genotype patients increased incidence of major eye events, stroke, and all cerebrovascular events, and aggressive antihypertensive treatment for CC genotype patients nearly reduced the risk of all cerebrovascular events. This suggests that CC genotype carriers were at high risk for T2DM with cerebrovascular events and should be actively treated with antihypertensive therapy. Nonetheless, these CC genotype patients presented a lower risk for concomitant coronary heart disease. Functionally,in myocardial tissue, *RASGRP1* mediates angiotensin П-induced phosphorylation of p38 MAPK and expression of periostin protein [19], inhibiting the expression of *RASGRP1* in the heart may help improve heart failure. Conversely, in brain tissue, phospholipase-c gamma activates Ras on golgi through *RASGRP1* to induce the differentiation of nerve cells, the transformation of fibroblasts and radiation resistance and other physiological processes [28], and *RASGRP1* is beneficial for brain function. This indicates the tissue-specific role of *RASGRP1*, emphasizing that reasonable intervention in patients with different genotypes of rs7403531 contributes to the long-term prognosis of the disease.

In conclusion, our primary findings included: 1) *RASGRP1* rs56254815 polymorphism was associated with antihypertensive efficacy. Compared with AA genotype carriers, AG/GG genotype T2DM patients had a higher blood pressure, and these patients showed better efficacy in antihypertensive therapy. 2) rs7403531 CC genotype patients were more likely to cause major eye diseases, cerebrovascular events, and stroke events, while coronary heart disease events are less likely to occur. For CC genotype patients, moderate antihypertensive treatment can be conducted. 3) Intensive hypoglycemic therapy could reduce major microvascular events, including major nephropathy. 4) rs7403531 T allele could reduce the risk of major microvascular events and major eye diseases, but intensive hypoglycemic lowering can increase the risk of overall vascular events in such carriers. Our findings may provide novel insight for personalized treatment for T2DM patients in clinical. However, the specific mechanism of *RASGRP1* genetic variation that affecting vascular complications in T2DM patients remains to be studied.

**Compliance with Ethical Standards**

**Declarations of interests** All authors declare no conflict of interest relevant to the subject matter or materials discussed in the article

**Ethics approval and consent to participate** Not applicable

**Availability of data and materials** Not applicable

**Contribution statement**

Jiecan Zhou, Fazhong He, Wei Zhang were responsible for the study design. Jiecan Zhou, Fazhong He, Bao Sun were responsible for the experiments. Jiecan Zhou, Fazhong He, Bao Sun did the data processing and statistical analysis. Jiecan Zhou, Bo Xu, Fazhong He, Yan Shu wrote and interpreted the paper. Zhaoqian Liu, Xiaoping Chen, Wei Zhang were responsible for data management and contributed reagents and materials.

**Funding**

This study was supported by the NSFC (82003872), Natural Science Foundation of Hunan Province (2022JJ50163, 2023JJ20034), Scientific Research Fund Project of Hunan Provincial Health Commission (20201973), Central government funds for guiding local scientific and Technological Development (2021QZY016), Hunan Province Clinical Medical Technology Innovation Guidance Project (2020SK51823, 2021SK51828, 2021SK51823), Hunan Provincial Clinical Medical Research Center for Drug Evaluation of major chronic diseases (2023SK4040). The Fund Project of University of South China (2020-25 and 2020-26), The Fund Project of Hengyang city (2020hcjz6713, 2020hcjz6716). The science and technology innovation Program of Hunan Province (2023RC3173).

**References**

[1] Meier JJ, Bonadonna RC. Role of reduced beta-cell mass versus impaired beta-cell function in the pathogenesis of type 2 diabetes. Diabetes Care. 2013, 36 Suppl 2:S113-119.

[2] Walaszczyk E, Luijten M, Spijkerman AMW, et al. DNA methylation markers associated with type 2 diabetes, fasting glucose and HbA1c levels: a systematic review and replication in a case-control sample of the Lifelines study. Diabetologia. 2018, 61:354-368.

[3] Rao Kondapally Seshasai S, Kaptoge S, Thompson A, et al. Diabetes mellitus, fasting glucose, and risk of cause-specific death. N Engl J Med. 2011, 364:829-841.

[4] Hivert MF, Vassy JL, Meigs JB. Susceptibility to type 2 diabetes mellitus--from genes to prevention. Nat Rev Endocrinol. 2014, 10:198-205.

[5] Fadista J, Vikman P, Laakso EO, et al. Global genomic and transcriptomic analysis of human pancreatic islets reveals novel genes influencing glucose metabolism. Proc Natl Acad Sci U S A. 2014, 111:13924-13929.

[6] Kwak SH, Park KS. Recent progress in genetic and epigenetic research on type 2 diabetes. Exp Mol Med. 2016, 48:e220.

[7] Odegaard JI, Chawla A. Pleiotropic actions of insulin resistance and inflammation in metabolic homeostasis. Science. 2013, 339:172-177.

[8] Donath MY, Shoelson SE. Type 2 diabetes as an inflammatory disease. Nat Rev Immunol. 2011, 11:98-107.

[9] Zhu J, Tseng YH, Kantor JD, et al. Interaction of the Ras-related protein associated with diabetes rad and the putative tumor metastasis suppressor NM23 provides a novel mechanism of GTPase regulation. Proc Natl Acad Sci U S A. 1999, 96:14911-14918.

[10] Ebinu JO, Bottorff DA, Chan EY, et al. RasGRP, a Ras guanyl nucleotide- releasing protein with calcium- and diacylglycerol-binding motifs. Science. 1998, 280:1082-1086.

[11] Kortum RL, Rouquette-Jazdanian AK, Samelson LE. Ras and extracellular signal-regulated kinase signaling in thymocytes and T cells. Trends Immunol. 2013, 34:259-268.

[12] Qu HQ, Grant SF, Bradfield JP, et al. Association of RASGRP1 with type 1 diabetes is revealed by combined follow-up of two genome-wide studies. J Med Genet. 2009, 46:553-554.

[13] Plagnol V, Howson JM, Smyth DJ, et al. Genome-wide association analysis of autoantibody positivity in type 1 diabetes cases. PLoS Genet. 2011, 7:e1002216.

[14] Yang HC, Li HW. Analysis of homozygosity disequilibrium using whole-genome sequencing data. BMC Proc. 2014, 8:S15.

[15] Taneera J, Lang S, Sharma A, et al. A systems genetics approach identifies genes and pathways for type 2 diabetes in human islets. Cell Metab. 2012, 16:122-134.

[16] Song C, Wang M, Fang H, et al. Effects of variants of 50 genes on diabetes risk among the Chinese population born in the early 1960s. J Diabetes. 2019.

[17] Li JY, Tao F, Wu XX, et al. Common RASGRP1 Gene Variants That Confer Risk of Type 2 Diabetes. Genet Test Mol Biomarkers. 2015, 19:439-443.

[18] Li H, Gan W, Lu L, et al. A genome-wide association study identifies GRK5 and RASGRP1 as type 2 diabetes loci in Chinese Hans. Diabetes. 2013, 62:291-298.

[19] Li L, Fan D, Wang C, et al. Angiotensin II increases periostin expression via Ras/p38 MAPK/CREB and ERK1/2/TGF-beta1 pathways in cardiac fibroblasts. Cardiovasc Res. 2011, 91:80-89.

[20] Ko B, Joshi LM, Cooke LL, et al. Phorbol ester stimulation of RasGRP1 regulates the sodium-chloride cotransporter by a PKC-independent pathway. Proc Natl Acad Sci U S A. 2007, 104:20120-20125.

[21] Ko B, Kamsteeg EJ, Cooke LL, et al. RasGRP1 stimulation enhances ubiquitination and endocytosis of the sodium-chloride cotransporter. Am J Physiol Renal Physiol. 2010, 299:F300-309.

[22] Scott RA, Freitag DF, Li L, et al. A genomic approach to therapeutic target validation identifies a glucose-lowering GLP1R variant protective for coronary heart disease. Sci Transl Med. 2016, 8:341ra376.

[23] He F, Shu Y, Wang X, et al. Intensive Glucose Control Reduces the Risk Effect of TRIB3, SMARCD3, and ATF6 Genetic Variation on Diabetic Vascular Complications. Front Pharmacol. 2018, 9:1422.

[24] He F, Liu M, Chen Z, et al. Assessment of Human Tribbles Homolog 3 Genetic Variation (rs2295490) Effects on Type 2 Diabetes Patients with Glucose Control and Blood Pressure Lowering Treatment. EBioMedicine. 2016, 13:181-189.

[25] Study rationale and design of ADVANCE: action in diabetes and vascular disease--preterax and diamicron MR controlled evaluation. Diabetologia. 2001, 44:1118-1120.

[26] Li P, Chen K, Nie Y, et al. Association of obesity with glucose, blood pressure, and lipid goals attainment in patients with concomitant diabetes and hypertension. Curr Med Res Opin. 2015, 31:1623-1631.

[27] Turner RC, Cull CA, Frighi V, et al. Glycemic control with diet, sulfonylurea, metformin, or insulin in patients with type 2 diabetes mellitus: progressive requirement for multiple therapies (UKPDS 49). UK Prospective Diabetes Study (UKPDS) Group. Jama. 1999, 281:2005-2012.

[28] Bivona TG, Perez De Castro I, Ahearn IM, et al. Phospholipase Cgamma activates Ras on the Golgi apparatus by means of RasGRP1. Nature. 2003, 424:694-698.

**Figure 1.** (A) The atypical "EF" pair of RASGRP1 binds to calcium ions with a diacylglycerol (DAG) binding domain (B) Overview of DNA sample collection and random grouping of participants

**Figure 2. Effect of RASGRP1 gene variation on HbA1c (%) level**

(A) (B) (C) were the responses of patients with T2DM to rs7403531, rs12593201 and rs56254815 under the enhanced hypoglycemic (left) and standard hypoglycemic (right) modes, respectively. Data were expressed as mean ±SEM, P values were estimated by the mixed linear model, and were adjusted according to baseline gender, age, course of disease, body mass index (BMI), different genotypes at the time of visit and drug dose.

**Figure 3. Effect of RASGRP1 (rs7403531) gene variation on cumulative risk at clinical endpoints according to glycemic control strategies**

(A)(B)(C)(D)(E)(G) display major microvascular events, major retinopathy events, minor retinopathy events, major nephropathy events, minor nephropathy events, all microvascular events, and all combined macroscopic and microscopic vascular events.The vertical line represents additional information about microvascular events (diabetic nephropathy and retinopathy) at 24 and 48 months of follow-up.The time of the event was recorded as the follow-up date.The therapeutic effects of RASGRP1 (rs7403531) CC and CT/TT genotypes (hazard ratio and p value) were treated with a backward LR survival-cox regression model, and the baseline data were corrected.

**Figure 4. Effect of RASGRP1 genetic variation on systolic blood pressure response during follow-up between perindopril/indapamide treatment group and placebo group**

(A) (B) (C) respectively represent the different responses of rs7403531, rs12593201 and rs56254815.Data were expressed as mean ±SEM, P values were estimated using a mixed linear model, and baseline gender, age, course of disease, body mass index (BMI), combined drugs and drug dose were corrected. The first 6 weeks were the elution period.

**Figure 5. Effect of RASGRP1(rs7403531) genetic variation on clinical outcomes of coronary heart disease during follow-up between perindopril/indapamide treatment group and placebo group.**

The P values were estimated by the backward LR survival-cox regression model and the baseline data were corrected.(A)(B)(C)(D)(E)(F) represent the cumulative incidence of all cerebrovascular events, major cerebrovascular events, all stroke events, all coronary heart disease events, new or worsening renal events, and new or worsening eye events, respectively.

Supplementary Figure 1. Linkage imbalance diagram of candidate SNPs

(A) The linkage imbalance of rs56254815, rs7403531 and rs12593201 in 1000 gene database; (B) Linkage imbalance diagram of 1357 samples in this study after genotyping of rs56254815, rs7403531 and rs12593201 by SNPscanTM technique.
